# Priority Criteria for Community-Based Care Resource Allocation for Health Equity: Socioeconomic Status and Demographic Characteristics in the Multicriteria Decision-Making Method

**DOI:** 10.3390/healthcare10071358

**Published:** 2022-07-21

**Authors:** Hui-Ching Wu

**Affiliations:** 1 Department of Medical Sociology and Social Work, Chung Shan Medical University, Taichung 40201, Taiwan; graciewu@csmu.edu.tw; Tel.: +886-424-730-022 (ext. 12137); 2 Social Service Section, Chung Shan Medical University Hospital, Taichung 40201, Taiwan

**Keywords:** community-based care, resource allocation, health equity, multicriteria decision making, health policy, policymaking, SDG 10

## Abstract

SDG 10 stipulates that inequality within and between countries can be reduced by governmental policies that focus on the allocation of fiscal resources and social protection strategies to improve equity. The sustainability of community-based care stations is a crucial support network for achieving the goal of active aging. Unequal allocation would occur only if the populations of administrative districts are considered. Comprehensive policies, in accordance with data and sustainable goals, must consider multiple factors. Hence, this study used multicriteria decision making (MCDM) to investigate how nine criteria-related socioeconomic statuses (SES) and demographic characteristics are prioritized in community resource and funding allocation. Thirty-four community care and aging experts were invited to complete a questionnaire based on the modified Delphi method and the analytical hierarchy process (AHP) method. The assessment criteria for the allocation of community resources are prioritized in the following order: disability level, age, household composition, identity of social welfare, family income, ethnicity, marital status, educational attainment, and gender. Quantitative indices can be used to determine the importance of resource allocation policymaking. The benefit of this study lies in decision makers’ application of ranking and weighting values in public funding allocation ratios for community-based care resources for health equity in Taiwan.

## 1. Introduction

### 1.1. Active Aging, SDG 10, and Health Equity

In 2002, the World Health Organization proposed its active aging policy framework [1], highlighting active aging as a process of guiding aging through policies, encouraging older adults’ optimal pursuit of health and social participation, and providing them opportunities for a safe environment to effectively promote their quality of life. Therefore, active aging is important in that it allows older adults to achieve a state of successful aging. According to Phelan et al. [2], older adults believe that successful aging includes the integration of physical, functional, psychological, and social factors in multiple aspects of health conditions. To improve older adults’ quality of life, in addition to medical services, social activities must improve their mental flexibility, and connecting with a support network can help enhance their health. In older adults, age discrimination is associated with unhealthy results and dissatisfaction. Active aging is a multidimensional concept described by a set of characteristics, particularly health, positive mood, and control [3]. Active ageing at an individual level is conditioned by health, education, and good financial conditions [4]. Active aging policies have financial and economic implications and affect both men and women [5]. Inequality in active aging outcomes is critical for the design of appropriate and effective social policies [6]. This highlights the need for sustainable community-based care stations as a support network to achieve active aging.

In the discussion of resource allocation issues related to government funding, the most applied method is distribution by administrative districts in Taiwan. For fiscal restraint by the government and given the increasing age of the population, the provision of social welfare resources may need to be reduced. This would lead to unequal distribution, as it considers only the number of administrative districts in Taiwan [7]. The most deliberate and comprehensive policies, in accordance with data and long-term sustainable goals, must consider this scenario.

In Taiwan’s community care policy, active aging is designed for healthy older adults, and aging in place is guiding long-term care policies for disabled older adults in Taiwan [7]. For example, the number of community care stations is much lower than that of villages in 2021 [8], which affects older adults’ access to resources. Therefore, the researchers [9,10,11,12,13,14] suggested that a fair allocation of resources through resource optimization, which considers spatial and nonspatial factors, would help reduce inequities in community health.

The World Health Organization’s action guideline [15] is “health equity is defined as the absence of unfair and avoidable or remediable differences in health among population groups defined socially, economically, demographically or geographically”. Health equity means the absence of unjust and avoidable health differences among subgroups of the population. Equality is aimed at providing everyone with the same treatment, whereas equity involves giving people the resources they need to achieve the best health. People’s inequality represents an unequal distribution of social factors determining social health in society. Therefore, it is essential to identify health inequalities and their causes in order to achieve health equity [16].

The degree of health equality and access to equitable health care can be determined by factors such as proximity to health care service points, equity in access to health facilities, or equity in the achievement of health results [17]. Equity in access to health care is essential to measure health equity in community health policy [18,19,20,21,22]. Strengthening community support, improving resource access, and designing resource allocation policies aimed at health equity are tools to achieve the objective of aging in place [18,20,21,23,24]. For this reason, sustainable community-based care stations could be the essential support network for achieving health equity and active aging.

According to SDG 10 [25], reducing inequality within and between countries requires governmental policies, especially those that focus on the allocation of fiscal resources and enforce social protection policies to improve equity. Specifically, SDG 10.2 promotes the social, economic, and political inclusion and empowerment of everyone regardless of their age, sex, disability, race, ethnicity, origin, religion, or economic or other status. Meanwhile, SDG 10.3 ensures equal opportunity and reduces outcome inequalities by eliminating discriminatory laws, policies, and practices and by promoting appropriate legislation, policies, and action in this regard. Finally, SDG 10.4 focuses on fiscal, wage, and social protection policies and the progressive achievement of greater equality.

The evaluation of resource allocation decisions in healthcare could be based on two broad criteria: efficiency and equity [26,27,28,29]. Efficiency is a general measure of the production of a specific output with the least amount or quantity of waste, expense, or unnecessary effort [30]. In our study, efficiency could be described as gauging the capability to allocate resources to older adults with minimal access time to community care stations, therefore achieving minimum accessibility and equal allocation based on the average. Meanwhile, equity measures differences in the community care resources allocated to community stations in different districts based on their demographic and socioeconomic factors.

These two factors can often conflict and lead to different allocation policies [26,28,29]. For example, if each district’s “eligibility” is simply determined by their population, then achieving equity (i.e., minimizing inequality) would require all the people who live in these districts to have the same opportunity to be admitted. However, the population with lower living resources (e.g., low socioeconomic status) must be given priority if the objective is to maximize the efficiency of the system. Efficiency is commonly defined as a measure of the total utility incurred by resource recipients. Therefore, many assessments could be used to represent this general form of “efficiency”. For this reason, considerations regarding equity arise in many resource allocation contexts and also require different interpretations and rationales. The evaluation of the equity of community-based care resources affects public budget policy through fair resource allocation criteria, a reduction in regional resource inequality, and the provision of capital for the sustainable development of community-based care.

### 1.2. Multicriteria Decision Making (MCDM): SES, Demographic Characteristics, and Community Healthcare Equity

The promotion of health empowers people, communities, and societies to take charge of their own well-being and quality of life. Socioeconomic status (SES) is usually measured by a certain level of education, income, or occupation or by a composite of these dimensions to create a total measure for a family or individual [31]. SES has been applied to a large concept, indicating that the equity of public policy is assessed by considering the position of people, families, households, census tracts, or other aggregates established through these measurements. Epidemiological and sociological research on health also generally uses educational attainment, income, and occupational position as a measure for the SES to investigate distinct influences on health outcomes [32,33,34]. The association between educational attainment and seniors’ health often represents the one-way impact of SES on health, as educational attainment is often completed before the onset of adult health problems, and the association between income and health represents a bidirectional influence [35]. The measure of SES on health in sociological and epidemiological studies is essential for discussing the relationship between SES and risk factors for health [36].

Demography has also been understood to include measures of participation in social, cultural, or political life. The WHO 2030 Agenda for Sustainable Development [37] indicates that health literacy is more than a personal resource: higher levels of health literacy within populations could yield social benefits as well. This health inequality is not accidental, as the poor are more likely to live, work, study, and play in environments that are harmful to health. Individual and household poverty prevents disadvantaged people from having full access to health resources. The disadvantaged populations often suffer greater multidimensional costs of illnesses. If vulnerable populations are empowered in early and sustained health-promoting actions, this could prevent acute and chronic conditions or promote active and curative treatments. Individuals with disadvantaged demographic characteristics (such as age, gender, marital status, ethnicity, household composition, disability level, and social welfare allowance) may often be more likely to develop physical and mental health conditions due to their disadvantaged work environment and poor financial abilities [32,33,35,36,38,39,40].

Investigating the relations between SES and demographic characteristics alongside health and well-being is an important issue for economic inequality. This could address calls to improve the social reporting of the links between SES and health, which often shows evidence of persistent differential health outcomes [32,33,34,35,36]. Health inequities are endemic in all regions worldwide, and disease rates are significantly higher among the poorest or most disadvantaged populations. If vulnerable populations are empowered to take early and sustained actions toward health promotion, they could prevent acute and chronic conditions as well as promote active and curative treatments.

This study implements a contextual approach to the influence of SES and demographic characteristics on health, as contextual measures of both refer to the interrelation between the individual and the environment [41]. Contextual approaches typically involve measures in ecological areas and can also adopt multilevel analyses. Contextual approaches to SES examine the social and economic conditions that influence individuals in a shared social environment. Therefore, this study selects nine factors (i.e., age, gender, marital status, educational attainment, ethnicity, household composition, disability level, family income, and social welfare identity) to represent the impact of SES and demographic characteristics on community resource allocation. Meanwhile, the statistical data of these factors could be acquired from open government data, so they could be applied to policymaking.

Quantitative indices can be used to determine the importance of resource allocation policymaking. For example, indices chosen by experts in different fields could consider public policy priorities. The combination of these indices results in multicriteria decision making (MCDM). MCDM refers to the process of determining the most feasible solutions to everyday problems based on established criteria [42,43,44,45]. MCDM seeks optimal results in complex scenarios that include various indicators, conflicting objectives, and criteria. MCDM methods are useful when several criteria are simultaneously considered. MCDM can be further categorized as multiobjective decision making, which focuses on design problems, and multiattribute decision making, which addresses material selection problems. An MCDM model ranks several alternatives and recommends the highest-ranked option to the decision maker. To construct an MCDM model, scholars often apply the Delphi method and the analytical hierarchy process (AHP) technique to evaluate the weights provided by experts.

Through the MCDM method, this study aims to prioritize crucial criteria for community care resource allocation to reduce public policy funding inequality among administrative districts. To the best of our knowledge, this is the first study to implement the MCDM approach in optimizing community resource allocation for policymaking based on different criteria.

## 2. Materials and Methods

### 2.1. Design and Setting

The study criteria were evaluated and scored using experts’ opinions, including specialists in community care or aging, workers from primary care institutions, and chief executive officers of community care stations. The MCDM method was then implemented by first applying the modified Delphi technique to the criteria, as determined by the literature review and three experts. In the first round, to design questionnaires on issues, 12 criteria were proposed according to the literature, including house ownership, traffic distance, occupation, age, gender, marital status, educational attainment, ethnicity, household composition, disability level, family income, and social welfare identity. In the second round, three experts were invited to express their opinion and score the priority based on the proposed criteria. The AHP technique was performed to calculate the final weights of the criteria after three review rounds by these three experts. Therefore, the results of the questionnaires in the second round were obtained. The selected nine criteria in the second-round questionnaire include: age, gender, marital status, educational attainment, ethnicity, household composition, disability level, family income, and social welfare identity. According to Taiwan’s community care policy [46], residents who are qualified to access the resources of community care stations should be 55 years of age or older. Therefore, some criteria-related population should describe the limitation of “age 55 or older”, such as age, gender, marital status, educational attainment, ethnicity, household composition, and disability level.

**Figure 1:**
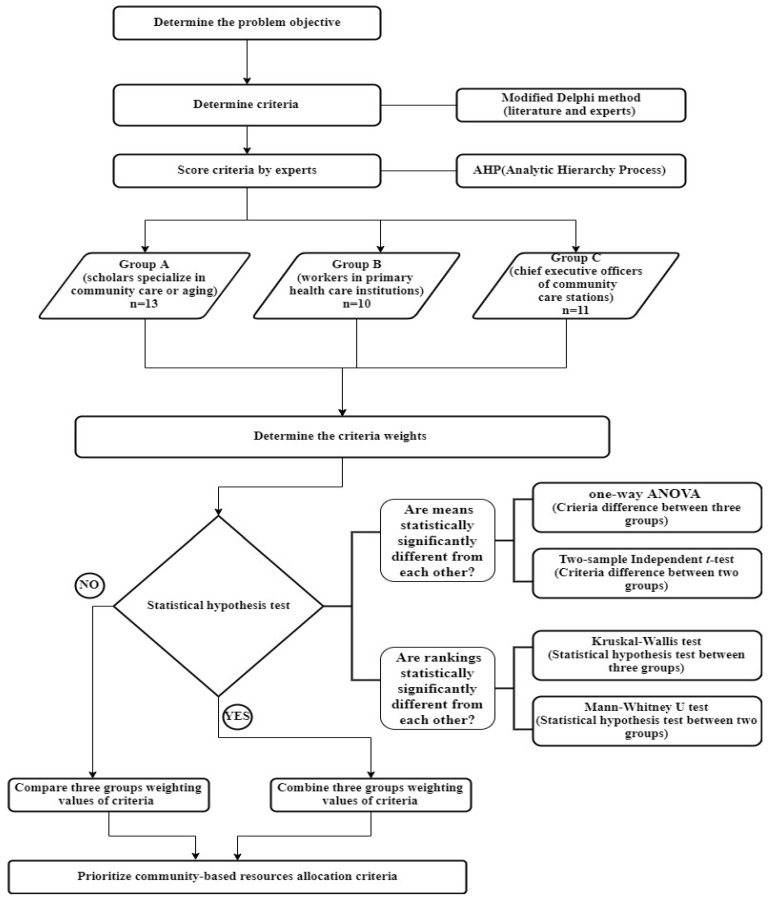
Research framework.

In the third round, the study recruited 34 experts from different fields to explore their approaches to prioritization. These experts were divided into three groups: (1) Group A: scholars specializing in community care or aging, such as professors in universities; (2) Group B: medical workers in primary healthcare institutions, such as registered professional nurses, physical therapists, speech therapists, and occupational therapists; (3) Group C: chief executive officers of community care stations, such as presidents and chiefs of staff. The experts in groups B and C were specialists in community care practice. To qualify the profession of these experts, this study invites experts based on the official government award roll “‘2022 Taiwan Community Care Golden Stations Award” [8]. Before determining the weights of the criteria, statistical hypothesis tests were performed to evaluate whether the weights of the three expert groups were statistically significantly different from each other. Finally, the weights were classified to prioritize community-based resource allocation criteria. The procedures are schematized in the research framework in Figure 1.

### 2.2. Modified Delphi Method

Policymaking refers to the selection of the best strategy among several alternatives. People’s diverse attitudes about the issues and factors that affect them generate multiplicity and sometimes conflicts among strategies. If decision making involves planning for the future, uncertainty often makes it more difficult.

The Delphi method is a series of sequence questionnaires or “rounds” mixed with controlled feedback whose goal is to reach a consensus among the most reliable opinions of the “expert group”. This method, developed mainly by Dalkey and Helmer [47] at Rand Corporation in the 1950s, is a widely used and accepted approach to collect data from respondents within their domain of expertise [48].

This technique was designed as a collective communication process aiming to integrate opinions on specific real-world issues, which includes conducting detailed investigations and discussions on specific topics for goal-setting, policymaking, or predicting future events. Delphi processes have been used in various fields such as program planning, needs assessments, policy decision making, and resource utilization to develop a wide range of alternative solutions. [49,50]. Delphi techniques are suitable for building consensus by collecting data from several questionnaires with multiple iterations. Instead of consensus, it focuses on identifying different views and answers through a debate process carried out through Delphi rounds [47,50,51,52,53].

The basic characteristics of the Delphi technique are as follows [54]: (1) Answers expressed through questionnaires and other communication cannot be traced to a specific panel member, promoting anonymity. (2) Several rounds of questions and controlled feedback allow for interactions to significantly reduce conflict among group members. Interaction is encouraged among members of a group in several phases. The results of the previous phase are summarized, and groups reevaluate their responses relative to the group’s thinking. (3) Statistical group responses are defined as the statistical averages of the final opinions of individual members. The final response reflects the views of all members.

The first-round implementation process of the traditional Delphi method only designs questionnaires on issues, invites experts to provide their opinions on specific matters, and allows experts to fully express their opinions to obtain a consensus on the issue in the first stage, and then the researchers analyze and aggregate these opinions [47,55,56]. Although the first-round process of the traditional Delphi method can prevent confrontation among experts, causing mutual interference, problems such as the prolonged time spent on questionnaire collection, the lower response rate of open questionnaires, and the excessive divergence of collected opinions can reduce the study’s credibility and validity [48,57]. A Delphi study involves distributing a sequence of customized questionnaires to a sample of experts until they reach a consensus or until their opinions are stable across questionnaire rounds. However, the definition of “consensus” in Delphi studies is a contentious issue. To achieve validity and reliability, a Delphi study must often involve at least four rounds to make sense of the consensus of the experts’ opinions. This makes for a costly undertaking in terms of both time and money for the researcher and the respondents.

Meanwhile, the modified Delphi method [48,57] captures the spirit and advantages of the original Delphi method and simplifies the complicated questionnaire process. The first round includes two modified methods: (1) Omit the open-ended consultation and do not use open-ended questionnaires to collect expert opinions. The modified Delphi method draws up various items based on the relevant literature or the researchers’ experience. Experts are asked to express their opinions based on the proposed items. This modification can reduce participants’ problems in answering open-ended questionnaires and improve the questionnaire recovery rate. (2) Combine the research process of the third round and fourth round and simplify the process into three stages. That is, send the results of the questionnaires in the second round to the experts, conduct a factor weight evaluation in the third round, and ask the experts to correct them according to the “importance” and “grade” of the classification elements in the questionnaire such that obtaining expert consensus and a stability of opinion would be easier.

Regarding the size of the expert group, Murry Jr and Hammons [48] believed that a sample of 10 to 30 in the revised Delphi method is preferred. Therefore, this study invited 34 experts (including scholars, workers, and chief executive officers specializing in community care or aging) to undergo questionnaires.

Delphi is not without its limitations. The consensus reached in a Delphi study may not be a true consensus and could be the product of specious or manipulated agreement. A specious consensus does not correspond to the best judgment and is instead a compromise. This is due to poor techniques in summarizing and presenting group responses and in ensuring common interpretations of the evaluation scales used in the research process [49].

Delphi data analysis could involve both qualitative and quantitative data. In classic Delphi studies, researchers must deal with qualitative data that use open-ended questions to seek the opinions of subjects, conducted in the initial iteration. Subsequent iterations identify and seek to achieve the desired level of consensus as well as identify any changes in judgments among panelists. Delphi studies often use measures of central tendency (means, median, and mode) and the level of dispersion (standard deviation and interquartile range) to present information regarding respondents’ collective judgments [58]. To achieve validity and reliability based on the modified Delphi method, this study includes the AHP technique [34,35] to implement the processes of the expert scoring criteria and determine the criteria weights.

In the modified Delphi method, after the expert questionnaires are collected in the second round, the AHP method simplifies the third round of complex expert consensus opinions. This process presents the spatial and nonspatial factors affecting the equitable allocation of community care resources and their weights.

### 2.3. AHP Method

The AHP method, developed by Saaty [59], is a measurement theory that adopts pairwise comparisons and relies on experts’ judgments to derive priority scales. It has been used for decision-making processes in different fields and has been adopted mainly in the discussion of decision-making problems under uncertainty and with multiple evaluation criteria. The strengths of this method include the simultaneous consideration of general attitudes and the detailed analysis in problem solving and the ability to account for the opinions of different experts. Since decisions may be inconsistent, the AHP method addresses measurement inconsistencies and improves judgments to the best possible extent to achieve better coherence [60]. The derivative priority scale is synthesized by adding the priority of the parent node to the parent node.

**Figure 2:**
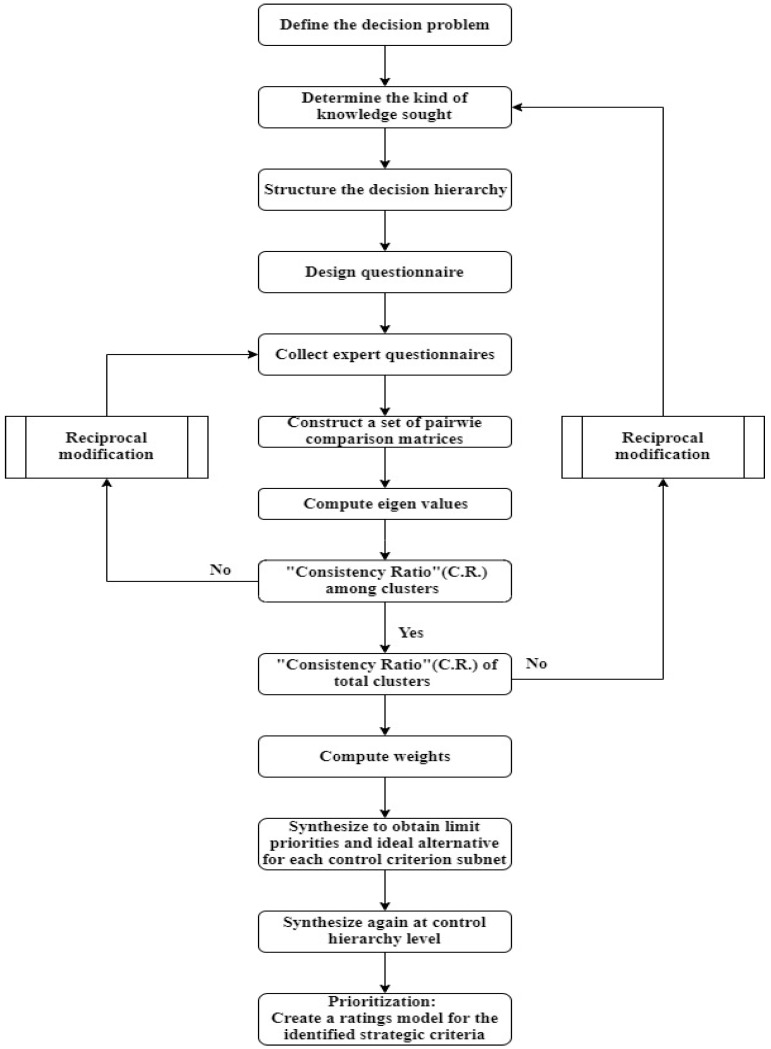
The AHP method.

Through quantitative techniques under AHP, complex problems can be systematically decomposed at different levels. The problem-solving steps using AHP are as follows (see Figure 2): (1) Describe the problem, identify the influencing factors, and establish a hierarchy, which could be described as “modeling”. (2) Using pairwise comparison and proportional scales, find the relative importance of the decision-making attributes at each level. Rate the criteria and calculate the relative weights among them or alternatives against different criteria. (3) Establish a pairwise comparison matrix, calculate its eigenvalues and eigenvectors, and obtain the weight of each attribute that is obtained. Use the consistency ratio (CR) to estimate the homogeneity of the judgments and the sensitivity analysis. A CR of less than or equal to 0.1 means that the expert’s opinion passed the consistency index test. Otherwise, continue with the “reciprocal modification” to confirm the logical consistency of the expert to achieve reliability.

**Figure 3:**
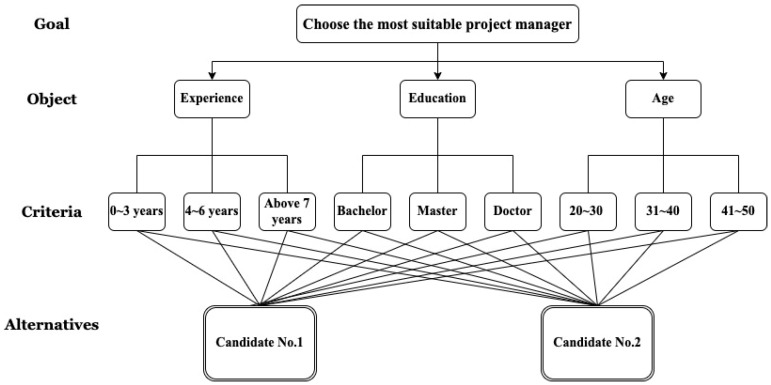
Decomposing the problem into a hierarchy.

The AHP method adopts the “decomposition” principle, which is divided into three layers from top to bottom (see Figure 3). For example, the problem could be choosing the most suitable project manager (“goal”), which is decomposed into the top “decision objective” (“objective”, such as experience, education, and age) and then in the sequence of the “decision criteria” (“criteria”) and the lowest level “alternatives” (“alternatives”, such as candidates). Through decomposition, a hierarchical diagram is formed to find each layer’s decision attributes of relative importance. The current research objective is to find the prioritization of criteria that could assess community resource allocation for health equity. Therefore, the decomposition of the problem does not need the “alternative” layer. The decomposition is thus divided into two layers from the top “objective” (defined as the “prioritization of criteria to assess the allocation of community resources for health equity”) and then in the sequence of “criteria” (defined as the nine main criteria: “age”, “gender”, “marital status”, “educational attainment”, “ethnicity”, “household composition”, “disability level”, “family income”, and “social welfare identity”). Using the AHP-based questionnaire, we invited experts to rate the importance of these criteria.

Regarding the AHP questionnaire scores, following the Saaty [60] evaluation scale, each evaluation criterion is divided into nine grades for a pairwise comparison. For instance, for the item “Which criterion do you think is relatively important for the allocation of community care resources?”, assuming that criterion A is set as “age” and that criterion B is set as “gender”, if the experts believe that “age” is relatively important, and such importance falls within seven points, then “7:1” is checked in the direction of “Criterion A is more important”. Table 1 shows the sample form of the AHP questionnaire.

Since expert opinions are likely to diverge on the principles for assessing resource allocation equity in community-based care, the application of the AHP method will help determine the most helpful decision-making factors to promote certainty. This study uses the expert opinions collected via the modified Delphi method, through the AHP method, to further define the weights of the influencing factors for evaluating the equity of community-based care resource allocation. The AHP method in this study is analyzed using Super Decisions [61], a software used for decision making with dependence and feedback. It implements the AHP and the analytic network process (ANP). This software provides tools to create and manage AHP and ANP models, enter judgments, obtain results, and perform sensitivity analysis on the results. It also supports complex, multilevel benefits–opportunities–costs–risks (BOCR) models.

This study applied pairwise AHP comparison models in Super Decisions to obtain weighting values and CR to explore prioritization between experts. The result of each expert questionnaire must pass the test standard (CR equal to or less than 0.1); otherwise, the questionnaire should be reciprocally revised to ensure the omission of logical errors.

A weighting of the representative importance of the criteria would be proposed, and then the priorities are listed based on rankings. The prioritization of community-based resource allocation would help government policy accurately assess resource-disadvantaged areas and distribute resources more equitably.

### 2.4. Statistical Hypothesis Test

Before determining the criteria weights, a statistical hypothesis test established whether the weights of the three expert groups differed statistically. If the result was statistically significant, the difference in opinions between the three expert groups would be compared. Otherwise, the results of the three groups would be combined to compute the weighting values and rank the priorities.

This study performed statistical hypothesis tests in means and rankings. For significantly different means, one-way analysis of variance (ANOVA) and the two-sample independent *t*-test were applied. For significantly different rankings, the Kruskal–Wallis test and Mann–Whitney U test were performed.

One-way ANOVA determines whether the mean values of three or more independent (nonrelated) groups are statistically significant [62,63,64]. For the weights based on the AHP method, the mean values underwent one-way ANOVA to test whether the importance of the criteria is different between the three groups.

The two-sample independent *t*-test compares the means of two independent groups to determine whether the means of the associated population are significantly different [65,66,67]. This study used this test to determine whether the main criteria were different between two groups.

The Kruskal–Wallis test is a rank-based nonparametric test for determining whether statistically significant differences exist between two or more groups of an independent variable on a continuous or ordinal dependent variable [68,69,70]. Therefore, this was used to identify whether the rankings were statistically different between three groups.

The Mann–Whitney U test compares differences between two independent groups when the dependent variable is ordinal or continuous but not normally distributed [71,72,73]. Accordingly, this test was applied to determine whether the rankings were statistically different between two groups.

The significance threshold was set at 0.01, which is all that is required.

## 3. Results

### 3.1. Participant Characteristics

First, this study invited three experts to review the first-round questionnaire. The final questionnaire adopted in this study achieved a consensus after three review rounds by these experts. Afterwards, to balance the points of view of different fields, 34 experts from three fields were recruited to complete the questionnaire based on the modified Delphi and AHP methods. Table 2 lists the criteria descriptions.

The experts in group A were scholars specializing in community care or aging. Those in group B were medical workers in primary healthcare institutions. Those in group C were chief executive officers of community care stations. Notably, the experts in groups B and C were recipients of the 2022 Taiwan Community Care Golden Stations Award [8]. As shown in Table 3, the experts were composed of 62% women and 38% men. More than 90% of the experts in groups A and C were older than 51 years, while the experts in group B were between the ages of 31 and 50. In terms of qualified working years, group A had more than 11 years of experience, and 90% of groups B and C had more than 6 years of experience. Therefore, all of the experts’ opinions in this study showed expert validity in assessing the priority of criteria in community resource allocation.

### 3.2. Evaluation of Community-Based Resource Allocation Criteria by Geometric Mean

For weights based on AHP, geometric mean values were applied, and the difference in criteria between the three expert groups was tested using one-way ANOVA. Table 4 presents the test results, showing no statistically significant differences between the three groups for each criterion.

For further discussion, this study applied the two-sample independent *t*-test to compare the means of two independent groups to determine whether there is statistical evidence that the mean values of the associated population are significantly different. Based on the results in Table 5, no significant differences were observed between the three groups.

### 3.3. Evaluation of Community-Based Resource Allocation Criteria by Ranking

This study adopted the ranking of the three groups’ weighting values in the statistical test to confirm whether differences in opinion existed.

First, the Kruskal–Wallis test was applied to identify statistically significant differences between the three groups. As shown in Table 6, no significant differences were observed between the three groups.

Furthermore, the weighting values were ranked between two expert groups using the Mann–Whitney U test, as shown in Table 7. No significant differences were found.

From the results in Table 4, Table 5, Table 6 and Table 7, these findings demonstrate that no statistical differences were found between the three expert groups. Hence, this study combined the geometric means of the three expert groups to calculate the weighting values.

Table 8 shows the prioritization of the assessment criteria for allocating community resources. The top five criteria are in the following order: disability level, age, household composition, social welfare identity, and family income. As suggested by the AHP method, an evaluation of more than seven criteria could complicate experts’ judgment [59,60]. This effect could also be observed from the weighting values of the nine criteria. The criteria ranking of the weighting values from sixth to eighth (including ethnicity, marital status, educational attainment, and gender) is less than 0.1, which means that these criteria have an impact, but it is not as strong as that of the top five. Thus, in this section, we will discuss the importance of the top five criteria in policy decision making on the allocation of community health resources for health equity.

## 4. Discussion

Investigating the relations between SES and demographic characteristics alongside health and well-being could improve the social reporting of the links between SES and health, which often shows evidence of persistent differential health outcomes [32,33,34,35,36]. The promotion of health empowers people, communities, and societies to take charge of their own well-being and quality of life. According to SDG 10 [25], reducing inequality within and between countries requires governmental policies, especially those that focus on the allocation of fiscal resources and enforce social protection policies to improve equity.

Through the MCDM method, this study aims to prioritize crucial criteria for community care resource allocation to reduce public policy funding inequality among administrative districts.

For the first-ranked criterion, disability level, which refers to the population aged 55 years or older distinguished by profound, severe, moderate, and mild disability, the population ratio should be considered seriously in community care resource allocation. People with disabilities often have a low employment rate, partly reflecting a history of social exclusion [74]. Enhancing the quality of community care for disabled older people could improve their independent living [75,76]. Therefore, the criterion for disability level is placed before age by experts in this study.

The third-ranked criterion, household composition, pertains to the population aged 55 years or older distinguished by living alone, living with one’s spouse, living with children, living with relatives and friends, and living in long-term-care institutions. Barriers to timely medical care for older people often consist of issues of disability status and household composition [77,78]. A link is observed between household composition and community social care dynamics. Research shows that the house composition of older women is different from that of the surviving children of women and that they are a unique potential provider in the household [79]. Older people are more likely to live in two- than in three-generation households. These issues recognize additional possibilities and problems of community care, such as living alone, living with other nuclear family relatives, and living in care institutions. The social isolation of older people in communities is often associated with differences in household composition. Mental health imbalances and the lack of social support from nonfamily members are associated with the risk of social isolation [74]. For those who live with their families, low cognitive activity and poor health practices are linked to social isolation risks. In this study, the experts evaluated household composition as the third most important criterion for the allocation of community care resources.

The fourth-ranked criterion is social welfare identity, which refers to households distinguished by low income and low–middle income. Considering economic-disadvantaged households, we emphasize the relation between social care and community care [80]. This criterion indicates that the status of each household’s welfare rights is critical to human well-being [81]. Therefore, households with an identity of social welfare, especially those at risk of poverty, depend on government subsidies for survival.

The fifth-ranked criterion is family income, which pertains to households’ annual income distinguished by percentile. For example, the quartile method is applied to classify households with the lowest 20% and the highest 20% annual incomes in the region, which could present regional wealth gaps that require funding to address poor services and the lack of resources. As Phillipson, Bernard, Phillips, and Ogg [78] proposed, investing in health could reduce poverty and focus on key policy areas for pro-poor health. All these goals could be achieved by promoting policy coherence in the fair distribution of community care resources.

The current discussion provides ample support to demonstrate the importance of these criteria in community care resource allocation in Taiwan. Compared with previous studies on the distribution of community care resources [9,10,11,12], this study strengthens the literature by prioritizing these criteria based on expert opinions and adopting the modified Delphi method and the AHP method. For government policy decision making, this study suggests applying weighting values to determine the allocation ratio of public funding in Taiwan community care policy.

## 5. Conclusions

SDG 10 [25] emphasizes the need for governmental policies to reduce inequality within and between countries, especially those that focus on the allocation of fiscal resources and enforce social protection policies to improve equity. In particular, SDG 10.2 promotes the social, economic, and political inclusion of everyone regardless of age, sex, disability, race, ethnicity, origin, religion, or economic or other status. SDG 10.3 ensures equal opportunity and reduces outcome inequalities by eliminating discriminatory laws, policies, and practices and promoting appropriate legislation, policies, and action. SDG 10.4 highlights the adoption of fiscal, wage, and social protection policies toward greater equality. Therefore, a resource allocation policy based on SES and demographic characteristics is necessary to achieve these SDG 10 goals and minimize health inequality.

Although 34 experts from three different fields were recruited, this study has certain limitations. The first concerns the number of criteria. As Saaty [59,60] suggested, evaluating more than seven criteria could be difficult for experts. Therefore, future research could circumspectly limit the criteria to seven. The second limitation is that, since the study invited only experts from Taiwan, the results could not be generalized to other countries.

For the criteria in this study to be the main criteria, many detailed subcriteria must be further assessed. Future research should investigate these subcriteria and further recognize their prioritization and weighting values. Therefore, such criteria evaluation would be more equal for the allocation of community-based care resources. Despite this study’s limitations, we hope that it could serve as a basis for policymaking in community-based care and health equity.

## Figures and Tables

**Table 1:** Example of the AHP questionnaire.

Criterion A	Is More Important ← Equally Important → Is More Important									Criterion B
	9:1	7:1	5:1	3:1	1:1	1:3	1:5	1:7	1:9	
Age		√								Gender

**Table 2:** Criteria descriptions.

Criteria	Description
Age	Population aged 55 or older
Gender	Population aged 55 or older distinguished by women and men.
Marital status	Population aged 55 or older distinguished by single, married, divorced, and spouse deceased.
Educational attainment	Population aged 55 or older distinguished by education degree.
Ethnicity	Population aged 55 or older distinguished by nonindigenous, indigenous, and immigrant.
Household composition	Population aged 55 or older distinguished by living alone, living with spouse, living with children, living with relatives and friends, and living in long-term-care institutions.
Disability level	Population aged 55 or older distinguished by profound, severe, moderate, and mild disability.
Family income	Households’ annual income distinguished by percentile.
Social welfare identity	Households distinguished by low income and low–middle income.

**Table 3:** Characteristics of Experts by Group.

Characteristics	Group A		Group B		Group C		Total	
Number of cases	13		10		11		34	
Gender								
Woman	6	46%	7	70%	8	73%	21	62%
Man	7	54%	3	30%	3	27%	13	38%
Age								
20–30 years	0	0%	1	10%	0	0%	1	3%
31–40 years	0	0%	5	50%	1	9%	6	18%
41–50 years	1	8%	4	40%	0	0%	5	15%
51–60 years	8	62%	0	0%	3	27%	11	32%
61 years and above	4	31%	0	0%	7	64%	11	32%
Work experience								
0–5 years	0	0%	1	10%	0	0%	1	3%
6–10 years	0	0%	5	50%	4	36%	9	26%
11 years and above	13	100%	4	40%	7	64%	24	71%

**Table 4:** Analysis of variance (ANOVA) by geometric mean values between the three groups.

	Group	Group A		Group B		Group C		*p*-Value
Criteria		Geometric Mean	Geometric SD	Geometric Mean	Geometric SD	Geometric Mean	Geometric SD	
Age		0.175	0.092	0.098	0.114	0.156	0.100	0.198
Gender		0.042	0.048	0.030	0.042	0.058	0.069	0.500
Marital status		0.058	0.027	0.038	0.048	0.070	0.055	0.258
Educational attainment		0.033	0.059	0.047	0.060	0.050	0.050	0.735
Ethnicity		0.047	0.074	0.083	0.084	0.053	0.055	0.467
Household composition		0.108	0.090	0.140	0.104	0.096	0.095	0.562
Disability level		0.173	0.110	0.156	0.093	0.116	0.136	0.477
Family income		0.074	0.105	0.080	0.092	0.092	0.064	0.885
Social welfare identity		0.067	0.095	0.106	0.088	0.081	0.103	0.628

**Table 5:** Two-sample independent t-test for the expert’s decision between the two groups.

	Group	*p*-Value		
Criteria		Group A vs. B	Group A vs. C	Group B vs. C
Age		0.087	0.099	0.229
Gender		0.012	0.511	0.281
Marital status		0.218	0.520	0.174
Educational attainment		0.581	0.459	0.902
Ethnicity		0.288	0.827	0.341
Household composition		0.438	0.754	0.324
Disability level		0.699	0.268	0.446
Family income		0.888	0.625	0.730
Social welfare identity		0.325	0.733	0.559

**Table 6:** Kruskal–Wallis test of criteria by expert groups.

	Values	Group A		Group B		Group C		*H* Statistic	*p*-Value
Criteria		Weighting Values	Ranking	Weighting Values	Ranking	Weighting Values	Ranking		
Age		0.225	1	0.126	9	0.202	3	0.213	0.899
Gender		0.054	24	0.039	27	0.075	18		
Marital status		0.075	19	0.049	25	0.091	16		
Educational attainment		0.042	26	0.060	22.5	0.065	21		
Ethnicity		0.060	22.5	0.107	12	0.069	20		
Household composition		0.139	7	0.180	5	0.124	10		
Disability level		0.223	2	0.201	4	0.150	6		
Family income		0.095	15	0.103	14	0.119	11		
Social welfare identity		0.086	17	0.136	8	0.105	13		

**Table 7:** Mann–Whitney U test of the main criteria by the rankings between two groups.

**A. Group A vs. Group B**								
	**Values**	**Group A**		**Group B**		***z*-Score**	**Standard Deviation**	***p*-Value**
**Criteria**		**Weighting Values**	**Ranking**	**Weighting Values**	**Ranking**			
Age		0.225	1	0.126	7	0.044	11.325	0.968
Gender		0.054	15	0.039	18			
Marital status		0.075	12	0.049	16			
Educational attainment		0.042	17	0.060	13.5			
Ethnicity		0.060	13.5	0.107	8			
Household composition		0.139	5	0.180	4			
Disability level		0.223	2	0.201	3			
Family income		0.095	10	0.103	9			
Social welfare identity		0.086	11	0.136	6			
**B. Group A vs. Group C**								
	**Values**	**Group A**		**Group C**		***z*-score**	**Standard Deviation**	***p*-value**
**Criteria**		**Weighting Values**	**Ranking**	**Weighting Values**	**Ranking**			
Age		0.225	1	0.202	3	0.530	11.325	0.596
Gender		0.054	17	0.075	12			
Marital status		0.075	13	0.091	10			
Educational attainment		0.042	18	0.065	15			
Ethnicity		0.060	16	0.069	14			
Household composition		0.139	5	0.124	6			
Disability level		0.223	2	0.150	4			
Family income		0.095	9	0.119	7			
Social welfare identity		0.086	11	0.105	8			
**C. Group B vs. Group C**								
	**Values**	**Group B**		**Group C**		***z*-score**	**Standard Deviation**	***p*-value**
**Criteria**		**Weighting Values**	**Ranking**	**Weighting Values**	**Ranking**			
Age		0.126	6	0.202	1	0.088	11.325	0.928
Gender		0.039	18	0.075	13			
Marital status		0.049	17	0.091	12			
Educational attainment		0.060	16	0.065	15			
Ethnicity		0.107	9	0.069	14			
Household composition		0.180	3	0.124	7			
Disability level		0.201	2	0.150	4			
Family income		0.103	11	0.119	8			
Social welfare identity		0.136	5	0.105	10			

**Table 8:** Priority of community-based resource allocation criteria.

	Values	Weighting Values	Ranking
Criteria			
Disability level		0.194	1
Age		0.186	2
Household composition		0.147	3
Social welfare identity		0.108	4
Family income		0.106	5
Ethnicity		0.076	6
Marital status		0.072	7
Educational attainment		0.055	8
Gender		0.055	8

## Data Availability

Not applicable.
